# Characteristic analyses of a neural differentiation model from iPSC-derived neuron according to morphology, physiology, and global gene expression pattern

**DOI:** 10.1038/s41598-017-12452-x

**Published:** 2017-09-25

**Authors:** Sai Kang, Xiaoxia Chen, Siyi Gong, Panpan Yu, SukYu Yau, Zhenghui Su, Libing Zhou, Jiandong Yu, Guangjin Pan, Lingling Shi

**Affiliations:** 1 Guangdong-Hongkong-Macau Institute of CNS Regeneration, Joint International Research Laboratory of CNS Regeneration, Ministry of Education of PRC, Jinan University, Guangzhou, China; 2 Department of Rehabilitation Sciences, Hong Kong Polytechnic University, Hung Hom, Hong Kong China; 3 Key Laboratory of Regenerative Biology, South China Institute for Stem Cell Biology and Regenerative Medicine, Guangzhou Institutes of Biomedicine and Health, Chinese Academy of Sciences, Guangzhou, China; 4 Co-innovation Center of Neuroregeneration, Nantong University, Nantong, China

## Abstract

Induced pluripotent stem cells (iPSCs) can differentiate into neural progenitor cells (NPC) under proper conditions. NPC can be used as a model and is a useful tool for disease mechanism exploration and drug screening. However, the characteristics of the cells in various stages from NPC to functional neurons have not been fully described. This study investigated the characteristics of iPSC-derived NPCs during differentiation. Morphological characteristics of the NPCs, including soma area, neurite length, and the number of neurite branches, were examined on selected differentiation days. Physiological functions were assessed by recordings of sodium current, spontaneous excitatory postsynaptic current (sEPSC), and spontaneous inhibitory postsynaptic current (sIPSC). Furthermore, gene expression patterns were assessed with RNA-seq. We found that NPCs derived from iPSCs can be differentiated into glutamatergic and gabaergic neurons. Cell growth peaked during differentiation day 7–12, as the soma area decreased after day 12, growth cone and the number of branches peaked at day 9 and decreased afterwards; whereas a functional synapse formed after day 23. RNA-seq analysis found that a differential expression pattern emerged by day 7. Overall, the study provides a framework for the differentiation process of hiPSC-derived NPCs.

## Introduction

Stem cells are thought to hold great potential for improving our understanding and thus for developing treatment for many diseases^1^. Takahashi and Yamanaka (2006) made a remarkable breakthrough in stem cell research when they generated ES-like cells from adult somatic cells using a cocktail of transcription factors^2–5^. More recently, new methods have been developed to reprogram adult somatic cells (such as fibroblasts) into pluripotent cells (iPSCs). This development has made it possible to generate patient-specific cells for the treatment of various diseases and disorders^6,7^. The advantage of patient-specific cells is that the cells could have the patient’s genetic background without any modification and are therefore not likely to be rejected by the immune system of the patients when transplanted. As iPSCs are derived from adult somatic cells, the ethical concerns of human embryo use do not apply. The possibility of creating neuronal cultures from human stem cells, particularly from human-induced pluripotent stem cells (hiPSC), originating from a patient, has received wide attention for the potential to create translatable disease-in-a-dish models. Following the discovery of iPSCs, several studies have fueled enthusiasm for their use in neurological disorders. Indeed, iPSCs from patients with neurological diseases—such as Alzheimer’s disease, Parkinson’s disease, and motor neuron disease—have been established successfully^8–19^. More importantly, previous studies have also shown that physiologically functional neurons, characterized by synaptic transmission and generation of action potentials, can be differentiated from iPSCs or fibroblast-direct conversion, indicating the neuronal cells induced from iPSCs are likely to be functional^20–27^. However, many limitations still affect the application of this technology in personalized medicine in a clinical setting. One of the main limitations is that the characteristic parameters of the differentiation cells in different stages have not been clearly described to date. In our study, we examined the transcriptome phenotype coupled with functional neuron mature process assessed by both morphology and electrophysiological analyses.

## Results

### *In vitro* neuronal progenitor cell model

**Figure 1:**
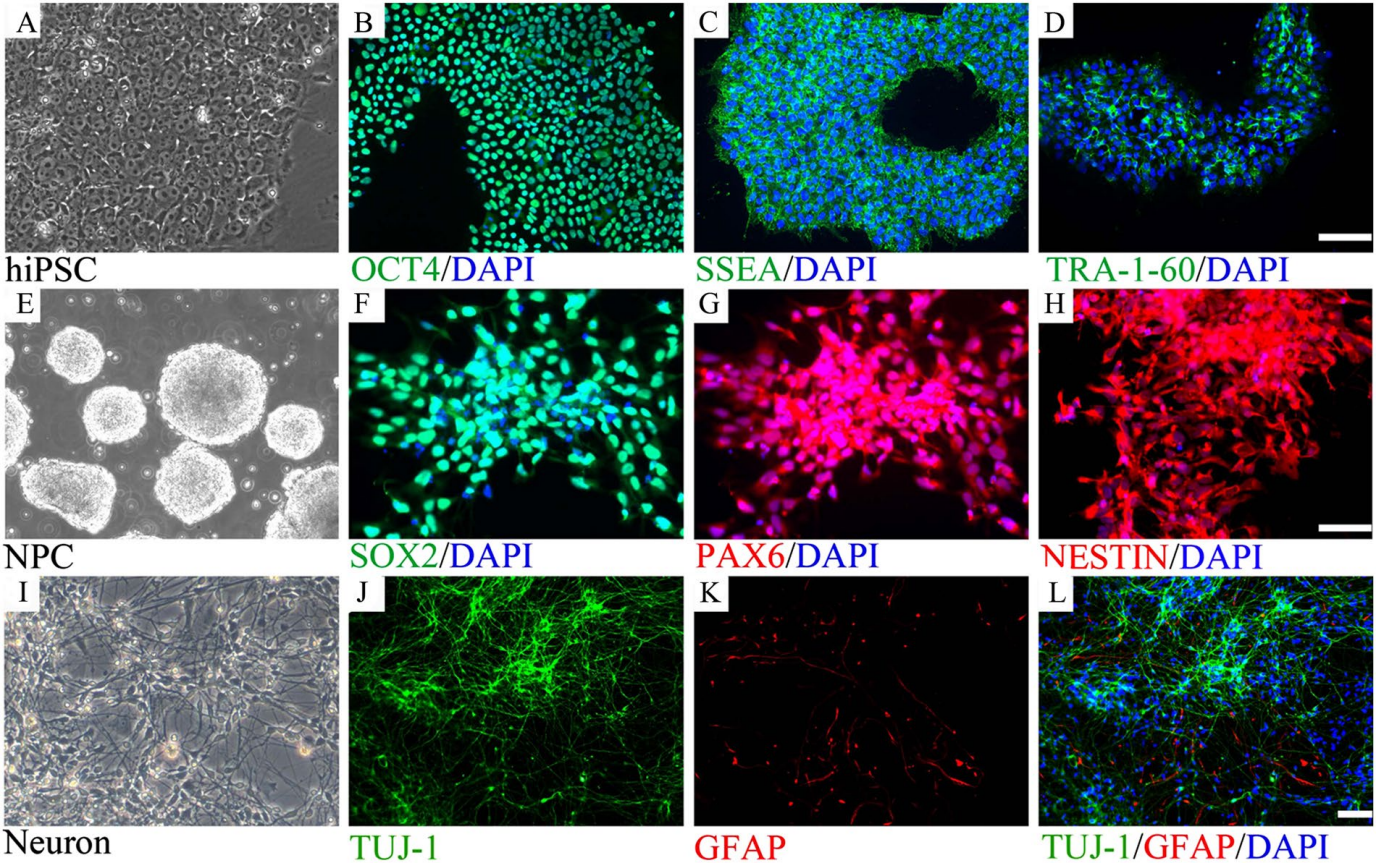
*In vitro* neural development model. Neural progenitor cells (NPCs) were differentiated from hiPSCs, which were then further induced to differentiate into neurons (**A**–**H**). The majority of cells differentiated from hiPSCs stained positive for NESTIN, indicating the cells were NPCs (**E**). NPCs derived from hiPSC maintained differentiation potential. HiPSC derived NPCs can diffentiated into both neural and glial lineage as stained by neuron marker TUJ-1, astrocyte marker GFAP (**I**–**L**).

We first established an *in vitro* neuronal progenitor cell (NPC) model by culturing hiPSCs with a two-inhibitor culture system. At the end of the culture period, the treated hiPSCs were stained for neuroectodermal stem cell markers including NESTIN, PAX6, and SOX2. We found that the majority of the treated cells stained positive for these markers, indicating that most of the treated hiPSCs differentiated into NPCs (Fig. 1).

We next examined the differentiation potential of these NPCs. The NPCs were cultured in a neuron differentiation media system (N_2_B_27_ + 20 ng bdnf + 1 μM dibutyryl-cAMP) for 21 days. The cells were then stained for TuJ1, a neuronal cell marker, and GFAP, an astrocyte marker. We found that both the neuronal marker and the astrocyte marker were expressed in the cultured cells (Fig. 1). These data indicated that NPCs derived from hiPSCs could differentiate into neuronal cells as well as astrocytes, and could be used as an in vitro model of neural differentiation. Furthermore, the neuronal cells stained positive for GABA, Glu1R, tyrosine hydroxylase (TH), and synapsin 1, indicating that the NPCs can differentiate into different types of mature neurons (Supplementary Fig. S1). Further analyses found that in differentiated cells, 54.9% were gabaergic neurons, 17.3% were TH-positive neurons, and 10.7% were glutamatergic neurons (Supplementary Fig. S1). The composition of neuronal cells did not change over the 15-day differentiation period.

### Neuronal growth profile

**Figure 2:**
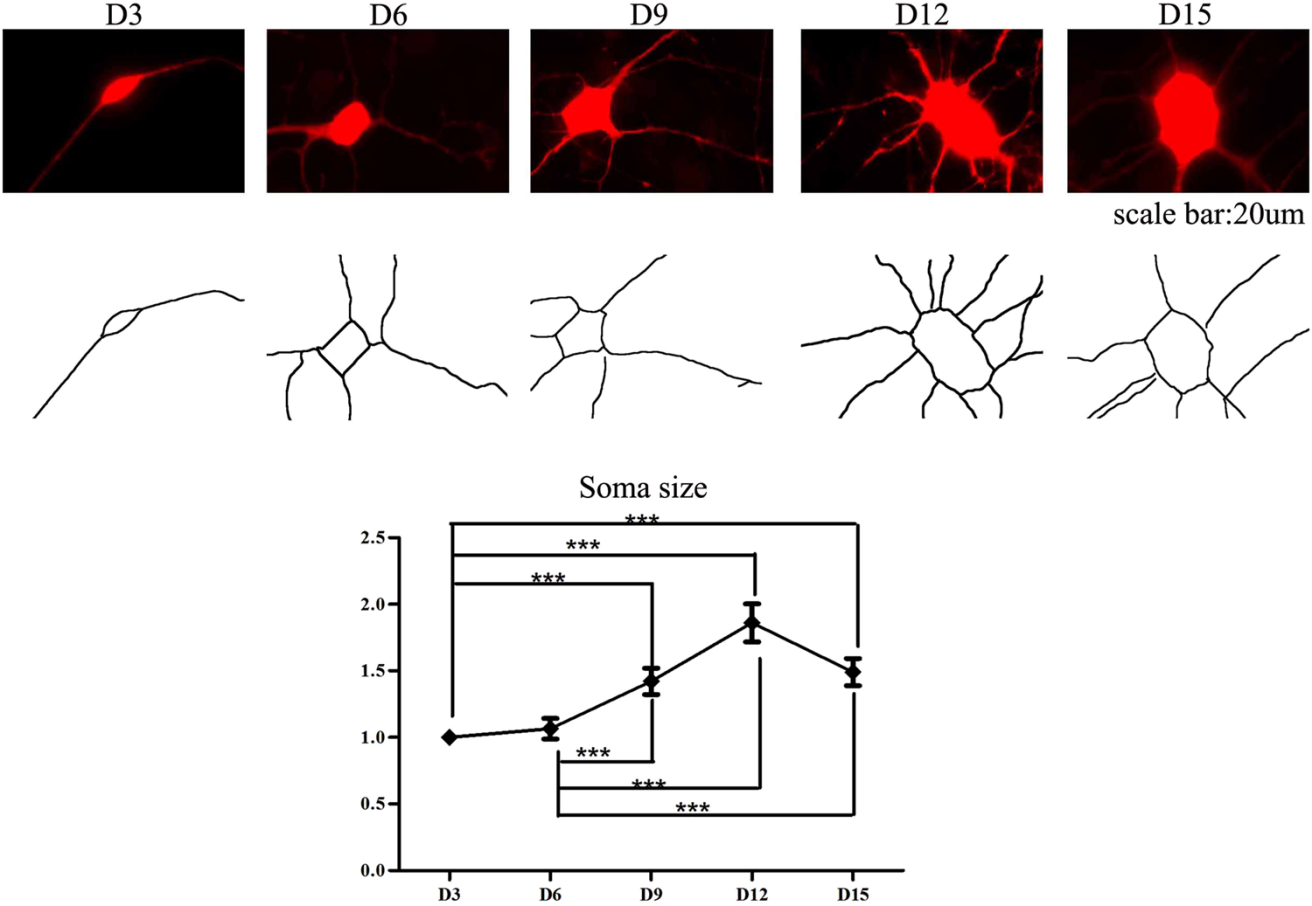
The area of soma increased during development. The soma area of the cultured neurons increased significantly when measured on D9, D12 and D15, compared with D3 (n = 60, ***P < 0.05).

**Figure 3:**
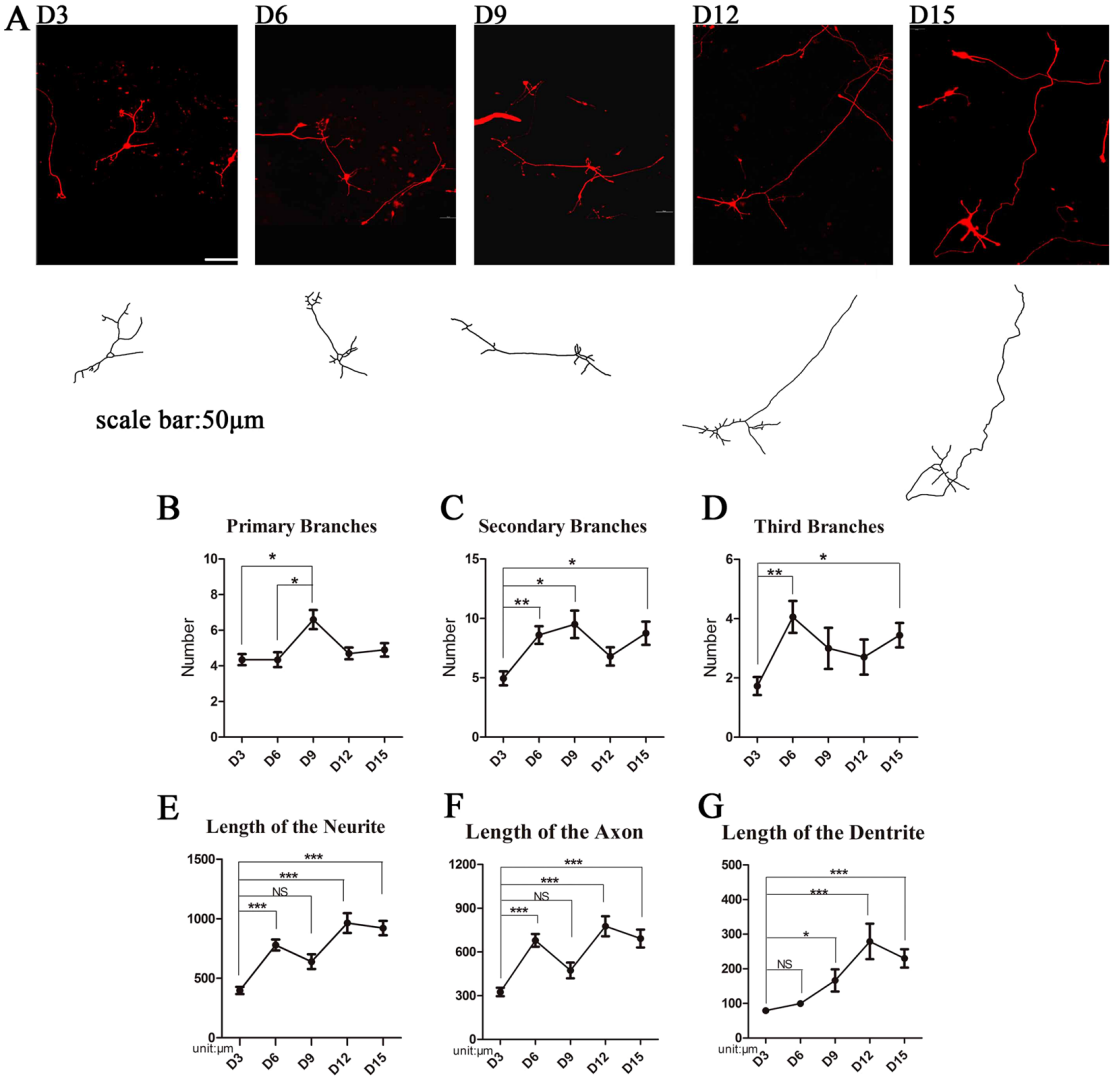
Both the length and the number of branches of neuritis increased during NPC maturation. (**A**) Cells expressing RFP were used for morphological study. The length of neuritis and the number of branches were measured and counted. (**B**–**D**) The number of primary and secondary branches of neurites reached a peak on D9. The number of primary branches showed a decreasing trend after D9. (**E**–**G**) The length of neurites reached a peak on D12 (n = 10 for D9, n = 20 for D3, D6, D12 and D15 measurement, ***P < 0.001, **P < 0.01, *P < 0.05).

We next investigated the morphological characteristics of these neurons. The somatic area of the neuronal cells and neurite length were measured, and the number of branches was counted in differentiated cells. The area of the somatic region increased significantly from D3 to D12. However, when assessed on D15, the somatic area decreased (Fig. 2). Time-lapse analysis showed that both the secondary and tertiary branches of the dendrites increased from D3 to D15, while the number of primary branches reached a peak on D9 of differentiation and then decreased to a level similar to that on D3 (Fig. 3). The lengths of the axons of the cultured neurons were significantly longer when measured on D9, D12, and D15 compared with the length on D3. We also noted that although the number of primary branches of dendrites reached a peak on D9, the length of the neurites continued to steadily increase throughout the 15 days of the differentiation protocol. However, the length of the neuritis did not increase significantly after D3, as no significant differences in neurite lengths were detected between neurons when assessed on D6, D9, D12, and D15 despite a trend toward an increase throughout the differentiation days. Overall, we found that the neurites of cultured neurons grow in length from D3 to D9; however, when assessed on later development days, both the area of the soma and the length of neurites decreased (Fig. 3).

**Figure 4:**
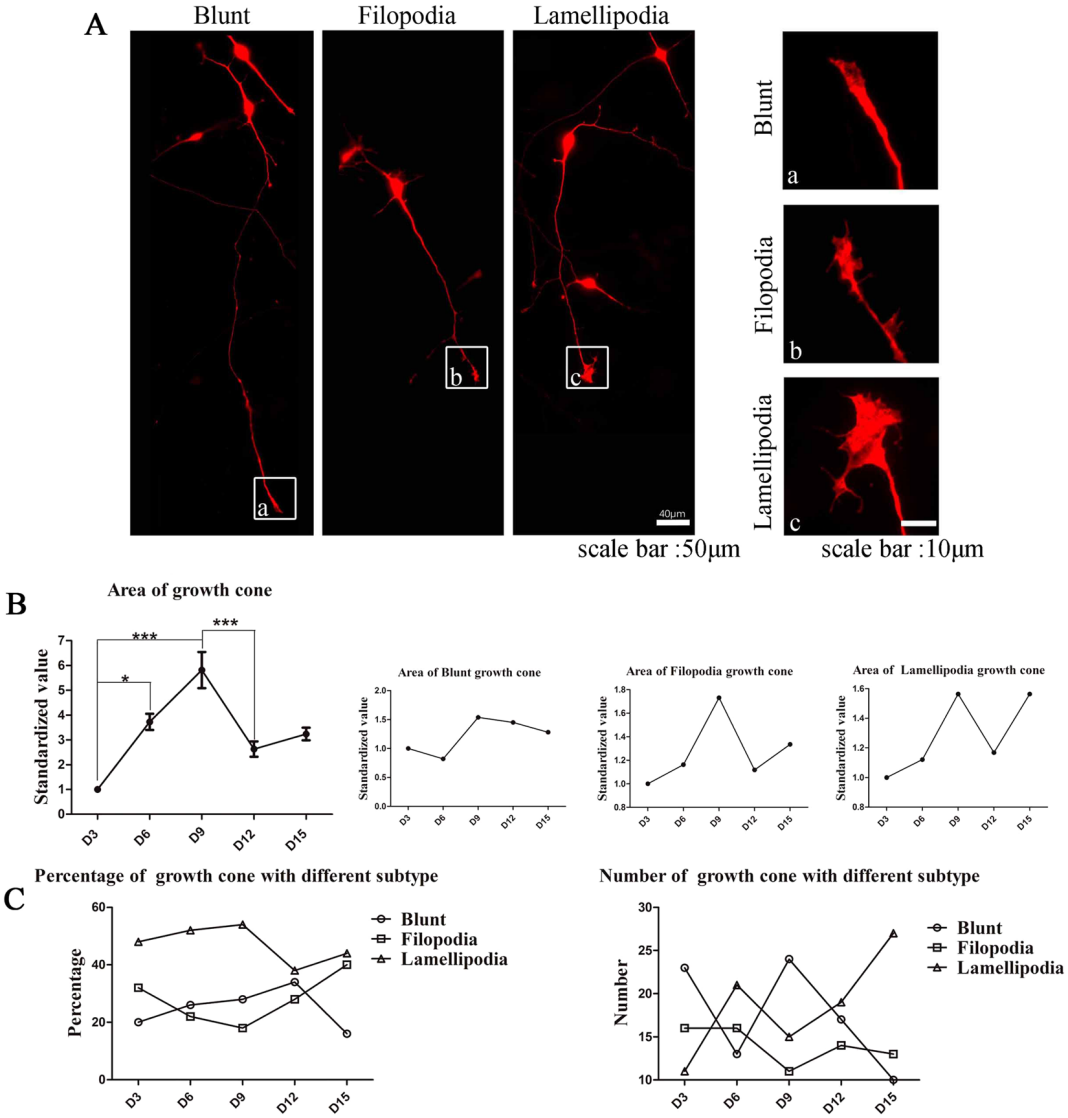
Development pattern of the growth cone. (**A**) The growth cones are categorized into three subtypes: blunt-ended (collapsed with no visible filopodia or lamellipodia), filopodial (growth cones with numerous filopodia and a small or absent lamellipodial veil), or lamellipodial (well spread growth cones with elaborate lamellipodia). (**B**) Area of growth cone reached a peak on D9. (**C**) The percentage of the lamellipodial type growth cone increased before D9 and then decreased (n = 60, ***P < 0.001, *P < 0.05).

We next characterized the development of the growth cone. The growth cones are categorized into three subtypes: blunt-ended (collapsed with no visible filopodia or lamellipodia), filopodial (growth cones with numerous filopodia and a small or absent lamellipodial veil), or lamellipodial (well spread growth cones with elaborate lamellipodia). The total growth cone area peaked on D9 and then decreased sharply (Fig. 4). In addition, we found that the ratio of the lamellipodia growth cone was the highest among the three subtypes during the 15-day differentiation period (Fig. 4).

### Electrophysiological profiles of differentiated neurons

**Figure 5:**
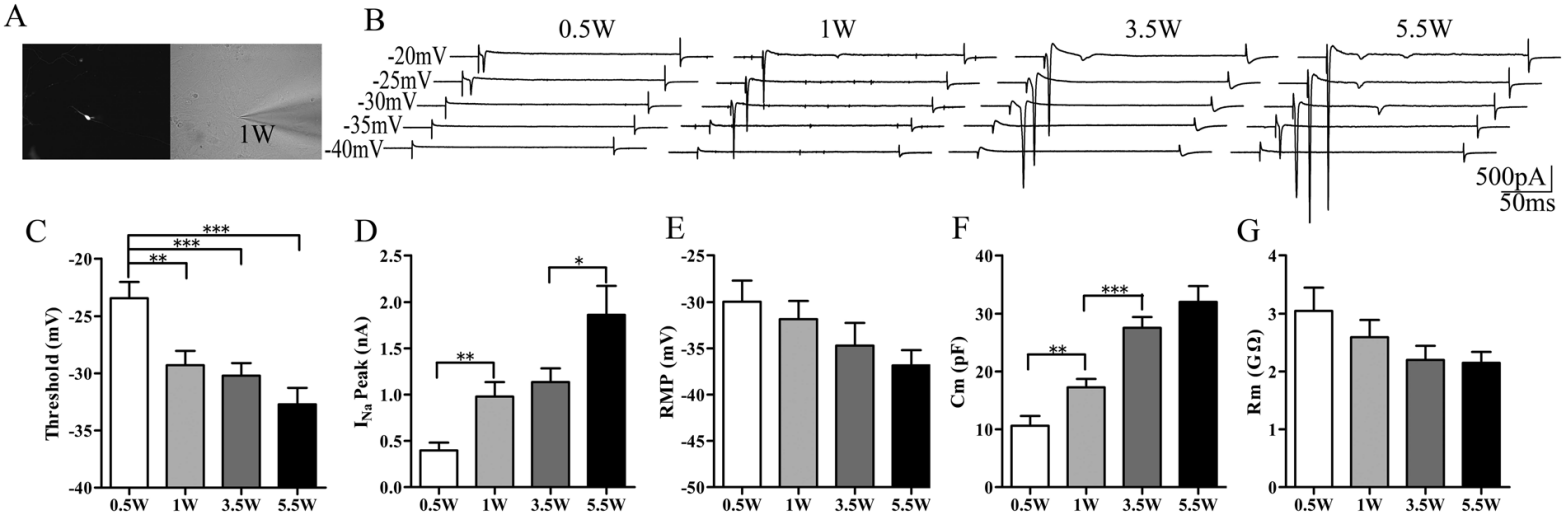
Sodium channels were expressed in early days of differentiation, but became functional only in later days. **(A**) RFP expressing cell visualized under microscope. (**B**) Sodium current traces recorded from cultured cells on different days during differentiation. (**C**) The depolarization that was required to elicit sodium current decreased. (**D**) The amplitude of sodium current increased. (**E**) The resting membrane potential of cultured cells decreased. (**F**) The capacitance of the cultured cells increased. (**G**) The membrane resistance of the cells decreased.

To test when iPSC-derived neurons have physiological activity, we performed whole cell recordings on RFP-expressing neurons at D4, D7, D24 and D38 (Fig. 5A). As shown in Fig. 5B, the sodium currents can be evoked by depolarizing steps. From D4 to D38, the threshold of evoking sodium currents shifted towards a more negative potential (Fig. 5C, D4 -23.4 ± 1.4 mV, n = 16; D7 -29.3 ± 1.3 mV, n = 28; D24 -30.2 ± 1.1 mV, n = 25; D38 -32.7 ± 1.4 mV, n = 24). In addition, the evoked sodium currents gradually increased during maturation (Fig. 5D, D4 -546 ± 91.6 pA, n = 16; D71048 ± 159.9 pA, n = 28; D24 1135.3 ± 148.2 pA, n = 25; D38 1862.2 ± 312.9 pA, n = 25). The resting membrane potentials shifted towards a more negative potential (RMP, Fig. 5E, D4 -30 ± 2.3 mV, n = 25; D7 -31.8 ± 2 mV, n = 29; D24 -34.7 ± 2.5 mV, n = 21; D38 -36.8 ± 1.6 mV, n = 26) and membrane resistances showed a decreasing trend (Rm, Fig. 5G, D4 3.05 ± 0.4 GΩ, n = 31; D72.59 ± 0.3 GΩ, n = 32; D24 2.2 ± 0.24 GΩ, n = 25; D38 2.15 ± 0.19 GΩ, n = 26). The membrane capacitance increased after differentiation was induced (Fig. 5F, D410.6 ± 1.7 pF, n = 31; D7 17.2 ± 1.5 pF, n = 32; D24 27.6 ± 1.9 pF, n = 25; D38 32 ± 2.7 pF, n = 26).

**Figure 6:**
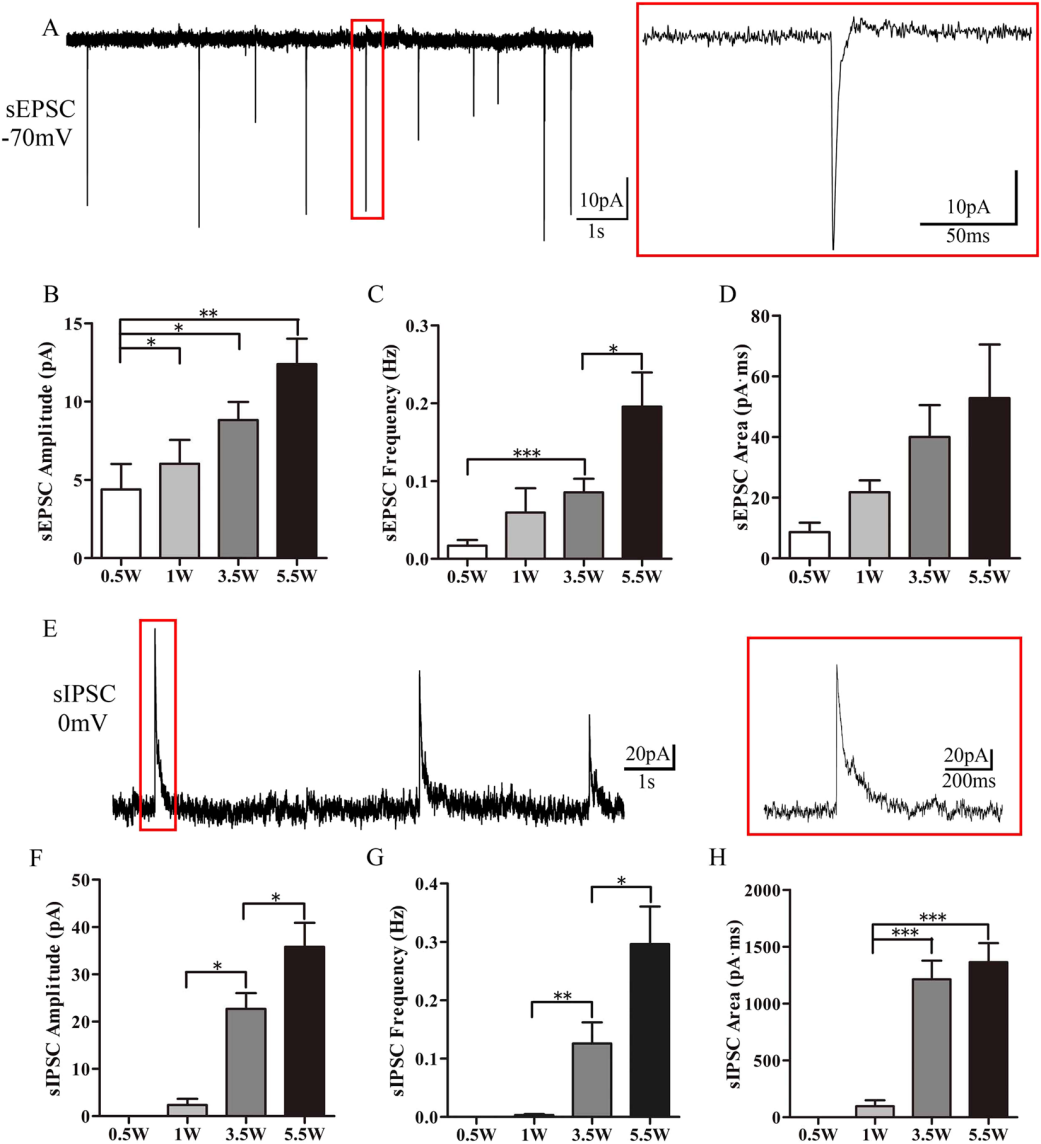
Frequency of sEPSCs and sIPSC progressively increased. (**A**) Example sEPSC traces recorded from a cultured cell. (**B**) The amplitude of the EPSCs increased. (**C**) The frequency of EPSC increased. (**D**) The charges transferred between cultured cells increased. (**E**) Example sIPSC traces recorded from a cultured cell. (**F**) The amplitude of sIPSC increased. (**G**) The frequency of sIPSC increased. (**H**) The charges transferred between cultured cells increased.

Next, we investigated whether functional synapses had developed in the iPSC-derived neurons by recording the synaptic events between neurons. Spontaneous excitatory postsynaptic currents (sEPSC) were recorded at all differentiation stages (Fig. 6A). The sEPSCs amplitude (Fig. 6B, D4 4.4 ± 1.6 pA, n = 21; D7 6 ± 1.5 pA, n = 28; D24 8.8 ± 1.2 pA, n = 25; D38 12.4 ± 1.6 pA, n = 25) and sEPSC frequency progressively increased (Fig. 6C, D4 0.017 ± 0.007 Hz, n = 21; D7 0.06 ± 0.031 Hz, n = 28; D24 0.086 ± 0.017 Hz, n = 25; D38 0.196 ± 0.044 Hz, n = 25) after differentiation was induced. The area of sEPSC did not increase significantly but showed an increasing trend (Fig. 6D, D4 8.64 ± 3.1 pA*ms, n = 21; D7 21.78 ± 3.91 pA*ms, n = 28; D24 39.99 ± 10.53 pA*ms, n = 25; D38 52.77 ± 25.81 pA*ms, n = 25). The glutamate receptor antagonist KyA (3 mM) blocked the sEPSC (data not shown). Spontaneous inhibitory postsynaptic current (sIPSC) could be recorded after 1 week of differentiation. Both the amplitudes (Fig. 6F, D4 0 ± 0 pA, n = 18; D7 2.4 ± 1.3 pA, n = 18; D24 22.7 ± 3.4 pA, n = 25; D38 35.8 ± 5.1 pA, n = 25) and frequencies (Fig. 6G, D4 0 ± 0 Hz, n = 18; D7 0.003 ± 0.002 Hz, n = 18; D24 0.12 ± 0.036 Hz, n = 25; D38 0.296 ± 0.064 Hz, n = 25) of the sIPSCs increased with time. Also, the area of the sIPSCs increased over time (Fig. 6H, D4 0 ± 0 pA*ms, n = 18; D7 97.4 ± 53 pA*ms, n = 18; D24 1214.1 ± 152.5 pA*ms, n = 25; D381364 ± 156.6 pA*ms, n = 25). The GABA receptor antagonist SR95531 (10 µM) completely blocked sIPSCs (data not shown).

### The pattern of gene expression during the course of differentiation

**Figure 7:**
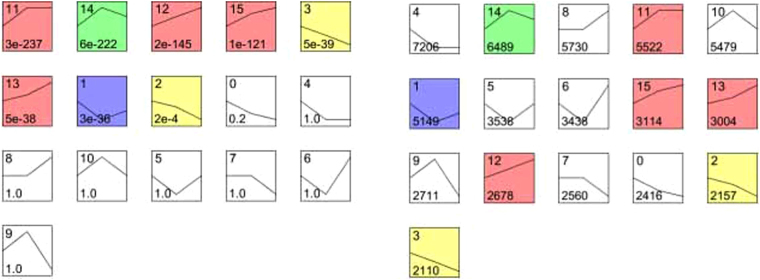
The enriched gene modules were divided into three groups by trend in expression. Squares with colors indicate a P value of <0.01. Four modules (red, No. 11, 12, 13, 15) belong to the increasing gene expression module, 2 modules (yellow, No. 2, 3) belong to the decreasing cluster, and the expression level of 2 modules (purple and green, No 1, 14) correlate with disorders.

We analyzed the gene expression profile of NPCs throughout the differentiation stages by time-lapse analysis using STEM software. We found 16 modules of genes including more than 30 000 genes that were enriched in NPCs. The 16 modules can be divided into three groups by trends of expression level. Of the 16 modules, 8 showed statistically a significant difference. The three groups were a decreasing (2 modules) group, increasing (4 modules) group, and disorder-associated (2 modules) group (Fig. 7).

Modules 11, 12, 13, and 15 showed a pattern of increasing expression, and GO pathway analysis showed that the increasing group of genes primarily included the genes that decided neuronal fate and the genes that are involved in the anatomic structure of neural cells morphology and function (Table S1). Specifically, module 11 included genes that regulate neuron projection and soma to dendritic compartmentalization, module 12 included genes that regulate cytoplasmic vesicle and extracellular structure organization, module 13 included genes that regulate ion channel activity and transmembrane transporter activity, and module 15 included genes that regulate potassium ion transmembrane transport, synaptic transmission.

We next annotated the enriched genes with KEGG in the early differentiation period from D0 to D7 (Table 1). From D0 to D7, the genes in the signaling pathways that regulate the general biological proliferation of “stem cells”, such as DNA replication, cell cycle, spliceosome, and others, were down regulated (Table 1), whereas the genes in the pathways that regulate neuronal maturity, including the MAPK signaling pathway, neuroactive ligand-receptor interaction, Wnt signaling pathway, and others were up-regulated (Table 1). The disorder groups were module 14 and module 1. In these groups, the modulate synapse and neuron projection in module 14 showed a pattern of increased expression from D0 to D28.

**Table 1:** Up and down regulated pathway enrichment by KEGG.

Rank	MapID	MapTitle	Pvalue	AdjustedPv
**Down regulated pathway from D0 to D7 enrichment by KEGG**				
1	map03010	Ribosome	3.20E-31	5.70E-29
2	map03040	Spliceosome	1.71E-16	1.52E-14
3	map03030	DNA replication	6.09E-13	3.61E-11
4	map03013	RNA transport	6.96E-12	2.38E-10
5	map04110	Cell cycle	8.01E-12	2.38E-10
**Up regulated pathway from D0 to D7 enrichment by KEGG**				
1	map04010	MAPK signaling pathway	2.18E-16	3.40E-14
2	map04020	Calcium signaling pathway	7.22E-15	3.75E-13
3	map04070	Phosphatidylinositolsignaling system	4.67E-13	1.21E-11
4	map04080	Neuroactive ligand-receptor interaction	4.99E-08	8.66E-07
5	map04144	Endocytosis	3.83E-06	5.44E-05
6	map04012	ErbB signaling pathway	9.47E-06	0.000123
7	map00562	Inositol phosphate metabolism	4.56E-05	0.000508
8	map04310	Wnt signaling pathway	0.000168	0.001751
**Down regulated pathway from D7 to D28 enrichment by KEGG**				
1	map04080	Neuroactive ligand-receptor interaction	0.00396	0.17822223
2	map00514	O-Mannosyl glycan biosynthesis	0.010899	0.18965783
3	map04020	Calcium signaling pathway	0.012644	0.18965783
4	map04310	Wnt signaling pathway	0.035705	0.362217316
**Up regulated pathway from D7 to D28 enrichment by KEGG**				
1	map04510	Focal adhesion	7.19E-20	7.76E-18
2	map04512	ECM-receptor interaction	9.55E-13	5.16E-11
3	map04610	Complement and coagulation cascades	0.00025	0.004491
4	map04350	TGF-beta signaling pathway	0.001724	0.023275
5	map04810	Regulation of actin cytoskeleton	0.013427	0.145012
6	map04340	Hedgehog signaling pathway	0.017364	0.170483
7	map04115	p53 signaling pathway	0.026175	0.22296
8	map04310	Wnt signaling pathway	0.026838	0.22296

It is worth noting that the genes in the WNT-associated pathways presented a unique pattern. The expression of this group of genes was not uniform, with some genes being upregulated during D0–D7, some genes being upregulated during D7–28, and some genes being down regulated (Table 2). We found that the group of genes that were up regulated during D0–7 and genes that were up regulated during D7–28 did not overlap; however, PRKCA was found to be upregulated during D0–7 and down regulated during D7–28.

**Table 2:** WNT pathway enrichment by KEGG.

Stage(Day)	pattern	involved genes
0–7	up	CXXC4, PPP3R1, NKD2, MAPK10, SIAH1, PRICKLE1, PRICKLE2, WNT10B, CSNK1E, NLK, MAPK9, WNT2B, PLCB1, CTBP2, PRKCB, PLCB4, RAC3, PRKCA, DAAM1, CAMK2G, CAMK2A, CAMK2B, PRKCG, PPP3CA, APC2, MAPK8, PORCN, NOTUM, BTRC, GSK3B, FRAT1, FRAT2, PRKACB, APC, PPP3CB, PPP3CC, WNT7A
7–28	up	NFATC4, FZD5, FZD6, CCND1, SFRP4, JUN, BAMBI, FZD8, WNT8B, WNT5A
7–28	down	PRKCA, NLK, WNT7A

**Figure 8:**
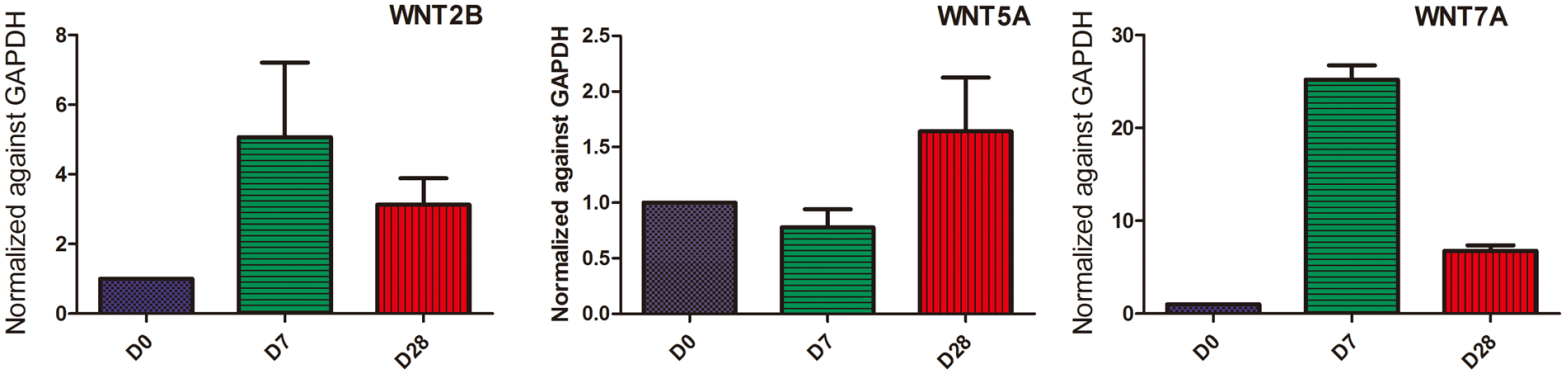
WNT signal pathway gene expression. WNT2B, WNT5A, and WNT7A gene expression were quantified by qRT-PCR. The results were consistent with the findings of RNA-Seq.

We further analyzed the expression pattern of WNTs (Supplementary Table S2). We found that WNT5A gene was down regulated, whereas WNT7A gene was up regulated in the early differentiation period (D0 to D7). In contrast, from D7 to D28, WNT5A gene was up regulated whereas WUT7A gene was down regulated. We then confirmed the expression of WNT2B, WNT5A, and WNT7A using qRT-PCR. The results were consistent with the findings when RNA-seq was used to assess gene expression (Fig. 8).

## Discussions

Since the development of the methods that make generating stem cells from adult somatic cells possible, the field of stem cell research has been fueled with the potential that these methods can offer. Of particular interest, stem cell therapies have shown promise in treating neurological disorders. However, the differentiation stages of the NPCs that are generated from iPSCs are not described in detail. In the present study, we analyzed the differentiation processes of hiPSC-derived NPCs using multiple analyses. We first assessed the morphological changes and then examined the physiological function of hiPSC-derived NPCs during cell maturation. Lastly, we examined the whole cell gene expression pattern.

We found that the growth of the somatic area of the hiPSC-derived NPCs followed an increasing and then decreasing pattern, in which the soma area increased significantly to day 12 and then plateaued. The growth of neurites followed a similar pattern, with the number of the primary, secondary, and tertiary branches peaking at differentiation day 3 to day 9 and the length of axons and dendrites peaking at differentiation day 9. The growth cone is the key factor of the guidance and motility of axonal growth in the early stage of differentiation and neuron regeneration^28^. Our study found that growth cones also showed a pattern of rapid growth up to differentiation day 9, followed by a significantly decrease. The bundled F-actin in blunt and filopodia growth cone transport cargo both anterogradely and retrogradely. In contrast, F-actin in the lamellipodium growth cone forms a dendritic network through the action of dendritic nucleator proteins and capping proteins. It is not surprising that the growth pattern of the growth cone coincides with that of the dendrites and the axon. Overall, morphologically, the growth of NPCs peaked at differentiation days 9 to 12, indicating that the cells at this stage have reached a stable differentiated stage.

The physiological functions of the NPCs were assessed with recordings of sodium current, sEPSC, and sIPSC. As the NPCs stained positive for Glu1, GABA, and synpasin 1, it is highly likely that the NPCs can signal through synapses with each other. Notably, sEPSC and sIPSC were both present in the NPCs. The amplitude and frequency of both EPSC and IPSC increased with cultured days, indicating the development of mature physiological function. This observation was consistent with previous findings that NPCs generated from iPSCs responded to glutamate or GABA stimulation after two months of neuronal differentiation^29^ and that sodium channel expression progressively increased in the iPSC-derived neurons, which eventually led to the generation of an action potential^30^. Overall, our results indicated that morphological maturation precedes functional maturation in these cells.

We next assessed the gene expression pattern over the differentiation stages and found that genes that are characteristic of “stem cells” were down-regulated, whereas genes in the neuronal cell induction pathway were up-regulated during differentiation day 0 to 7. These observations indicated that neural induction-associated pathways have emerged earlier in the timeline than the peak morphological changes and the development of physiological functions.

Of note, we observed that different genes in the WNT family present the opposite expression trend. WNT7A shows a significant increase in the early stage from D0 to D7, coupled with noncanonical Wnt signaling pathway genes, PRKCA and NLK; however, WNT5a shows a decreased expression pattern coupled with canonical Wnt signaling pathway genes FZD and β-Catenin in the early differentiation stage, which then increased. It was reported^31^ that the canonical Wnt signaling pathway is involved in regulating neuronal function maturation, while the noncanonical Wnt signalling pathway is related with cell fate and migration. In the present study, cell fate-related noncanonical Wnt signaling pathway emerged earlier than the neuronal function maturation-related canonical Wnt signaling pathway, which is in accordance with the development from fate to function.

Given the need for a model of iPSC-derived functional neuronal culture, our study confirmed that neuronal cells can be induced from iPSCs using a two-inhibitor induction system. The induced cells lost their pluripotency in the early differentiation stage. This study found that the method of developing neuronal cells from iPSC using a two-inhibitor induction system provides an in vitro model of valid neuronal differentiation. Using this model, we can perform drug screening on neuronal cells at the appropriate differentiation stages based on the morphological and physiological characteristics of the cells.

In the present study, we provide a framework for the differentiation process of hiPSC-derived NPCs. Compare to some researchers^19^, our neural differentiation model has higher effectiveness in converting iPSC into neuron and costs less time. The stage of NPC suspension culture can improve the purity and differentiation efficiency of neuron. Specially, we observed that functional synapse formed after day 23 by electrophysiological recording as well as a differential expression pattern emerged by day 7 by RNA-seq analysis, confirming by the expression of Wnt signaling pathway genes. Furthermore, neuron derived from patient iPSC can be used in drug screening and disease mechanism exploration, especially neurological diseases and neurodevelopment diseases, such as autism spectrum disorders, RETT syndrome, Alzheimer’s disease and schizophrenia. In 2013, Shcheglovitov^19^ proved the dysfunction of the ratio of excitation and inhibition neurons derived from PMDS patient iPSC and the synaptic deficits can be rescue by IGF1. What is more, translational research (such as cell transplantation) and genetic correction by CRISPR-Cas9 hold great promise for therapy of neurological diseases. Researchers had successfully corrected the defect gene form patient iPSC^32^, which can significantly promote the progress of efficient treatments for neurological diseases and neurodevelopment diseases.

## Methods

### Neuronal cell culture differentiated from stem cells

The hiPSCs clones (provided by CAS, Dr Pei’s lab) were cultured in mTeSR™ 1 medium (STEMCELL Technologies) in a matrigel (BD Matrigel™, hESC-qualified Matrix, 354277) coated dish. When the confluence of iPSCs in a well of a 6-well plate reached 95%, the cells were detached with EDTA (5 × 10^−4^ mol/L) and replated into one well of a 12-well plate. When the cells reached full confluence after 1–2 days, the medium was switch to N_2_B_27_ + 2 inhibitors (Dorsomorphin and SB4315242, Selleck) so as to induce neuronal differentiation. Cells were mechanical divided into fragments after 8 days of neuronal differentiation induction. The fragments in one well of 12 well plate were replanted into 2 wells of a 6-well matrigel-coated plate, and cultured in neural proliferation system I (N_2_B_27_, Thermo Fisher Scientific) medium. On the 8th day of proliferation, cell clones form one well of the 6-well plate were mechanical scraped into floating fragments, and the floating fragments were plated into non-coated T25 flask for floating culture with neural proliferation system II (N_2_B_27_ + 20 ng/mL bFGF + 20 ng/mL EGF, Thermo Fisher Scientific). Neurospheres usually formed on the second day of culture in neural proliferation system II. Accutase (StemPro® Accutase® Cell Dissociation Reagent, A1110501) was then used to detach neurosphere, and the single cells were plated in matrigel-coated dishes with neuron differentiation medium (N_2_, Thermo Fisher Scientific; B_27_, Thermo Fisher Scientific; 1 μM dibutyryl-cAMP, Sigma-Aldrich; 20 ng bdnf, PeproTech).

### Morphology analysis

The cultured cells were then infected with 5 mL of Lenti Virus (6 × 10^8^ TU/mL) packed with a tetracycline-controlled red fluorescent protein (RFP) expression sequence per well. The cells with RFP signals were then cultured at a density of 100 000 cells per well on a matrigel-coated coverslip that was placed at the bottom of a well of a 24-well plate for neuronal development for six hours, and then the vector was washed out. RFP expression was induced by addition of doxycycline to the medium after wash-off of the vector. The first day of induction on cover-slip was defined as day-0. Cells were used for morphological and functional analysis from D0.

Cells grown on coverslips were collected on culture day 0, 3, 6, 9, and 15; and their morphological characteristics were analyzed. The cells were fixed with 4% paraformaldehyde dissolved in phosphate based saline (PBS, pH 7.4) for 30 minutes. Cells were imaged with a microscope (Imager Z2, Zeiss) and the images were acquired with Zeiss camera (Axiocam 506 mono, Zeiss). The soma area, neurite length, and branches were analyzed with ImageJ 1.4.3.67 and NeuronJ 1.4.3. Statistic figure of T test was output by GraphPad Prism5.01.

*Below, D3, D7, D12, D15, and D21 indicate 3, 5, 12, 15, and 21 days from neurosphere culture, respectively.

### Growth cone analyses

Growth cone were categorized as described by Khazaei MR. Growth cones from 30 cells, collected from 20 coverslips, were visualized and analyzed with Image J.

### Electrophysiological recording

Cells were incubated at room temperature (25 ± 1 °C) in artificial cerebral spinal fluid (containing, in mM: 126 NaCl, 26 NaHCO_3_, 10 D-glucose, 2.5 KCl, 2 CaCl_2_, 2 MgCl_2_ and 1.25 NaH_2_PO_4_ pH 7.4) that was oxygenated with 95% O_2_ and 5% CO_2._ RFP-labeled cells were visualized with an IR-DIC microscope (Nikon Eclipse FN-1 microscope, 40X water objective). Whole-cell recordings were obtained with borosilicate microelectrodes (4–8 MΩ) pulled with an electrode puller that were filled with internal solution containing (in mM): 4 KCl, 126 K-gluconate, 10 HEPES, 0.3 Na_2_-ATP, 4 Mg-GTP, 10 phosphocreatine, pH 7.3, 290–310 mOsm. Eletrophysiological recordings were made with an amplifier (Axon MultiClamp 700B, Molecular Devices), signals were filtered at 3 kHz (low pass) and digitized at 20-kHz sampling frequency (DigiData 1550 A, Molecular Devices), and acquired and analyzed with pClamp10 (Molecular Devices). Neurons were first recorded in current-clamp mode, and the responses to 1 second current steps (−10 pA, 2 pA, or 3 pA steps) were recorded to determine the input resistance of the cells. Resting membrane potential (RMP) was not corrected for a liquid junction potential. Following current-clamp recording, neurons were recorded in voltage-clamp mode. The cells were hold at −70 mV, and the sodium currents were elicited by 5 mV voltage steps (200 ms) from -20 to 50 mV. Spontaneous excitatory postsynaptic currents (sEPSC) and spontaneous inhibitory postsynaptic currents (sIPSC) were recorded at holding potentials of −70 mV and 0 mV respectively. In some experiments, kynurenic acid (3 mM KyA, sigma) or SR95531 (10 µM, Tocris) was used to block sEPSCs or sIPSCs. The data were analyzed with Clampfit.

### Cell collection and mRNA preparation

Cells were collected at different time points. Total RNA was extracted using RNeasy mini kit, in combination with RNAase-free DNAase to remove the potential genomic DNA contamination (Qiagen). RNA concentration was quantified by Nanodrop 2000C Spectrophotometer.

### Gene expression validation by qRT-PCR

Total RNA was reverse transcribed into cDNA using the PrimeScript^TM^RT reagent Kit with gDNA Eraser (Perfect Real Time). Real-time quantitative PCR was performed with a SYBR® Premix Ex TaqTM II detection System. The primer sequences are shown as follows:

WNT2B: Forward: 5′-CCTGTAGCCAGGGTGAACTG-3′;

Reverse: 5′-CGGGCATCCTTAAGCCTCTT-3′.

WNT5A: Forward: 5′-GGGTGGGAACCAAGAAAAAT-3′;

Reverse: 5′-TGGAACCTACCCATCCCATA-3′.

WNT7A: Forward: 5′-TGGCTTCTCCTCAGTGGTAG-3′;

Reverse: 5′-CCTTCTCCTATGACGATGATGG-3′.

GAPDH: Forward: 5′-GATGTTCGTCATGGGTGTGAA-3′;

Reverse: 5′-AGTGATGGCATGGACTGTGGT-3′.

### Illumina transcriptome library preparation and sequencing

The total RNA purified from cells on neuronal differentiation day 0, day 7, and day 28 was subject to RNA-Seq analysis. RNA concentration was measured with Nanodrop One (ThermoScientific), and confirmed with Qubit 3.0 (invitrigon). The RNA integrity number was detected with BioAnalyzer 2100 (Agilent), Library construction was strictly followed Truseq RNA Access libarary Pre Kit (illumine, RS-301-2001), mRNAs were isolated from 400ng total RNA by PolyT capture beads in our lab protocol. The library length was detected by BioAnalyzer 2100 (Agilent), and the RNA concentration was confirmed by StepOnePlus™ Real-Time PCR System (Thermo fisher, 4376600). The mRNAs were sequenced with Hiseq X Ten (Illumina).

### Bioinformatic analysis

We used TopHat [49] version 2.0.13 for aligning the Illumina short reads against the reference human genome (Grch38.p7 http://www.gencodegenes.org/releases/25.html) as well as a reference GTF file constructed from Illumina’s iGenome annotation archive (http://cufflinks.cbcb.umd.edu/igenomeshtml, Homo Sapiens NCBI Grch38.p7). RESM (*RSEM: accurate transcript quantification from RNA-Seq data with or without a reference genome*)and edgeR (*edgeR: a Bioconductor package for differential expression analysis of digital gene expression data*.) were used to summarize the gene expression values as FPKM measures and to compare cell lines to identify genes with differential expression, separately. (Pvalue ≤ 0.05, FDR ≤ 0.001, fold change ≥2). Functional annotation for GO/KEGG was enriched by EnrichPipeline https://sourceforge.net/p/enrichmentpipeline/wiki/Home/STEM was used to do Time course cluster analysis (v1.3.9 *STEM: a tool for the analysis of short time series gene expression data*) Additionally, the results of gene expression (fold change and Pvalues) were overlaid with known protein-protein interactions^33,34^ in the Cytoscape^35^ software for network-based analysis and visualization.

### Statistical analysis

All data presented in the study are mean ± standard error unless otherwise noted. Between-group comparisons were tested with t-test or one-way ANOVA.

## Electronic supplementary material

Supplementary Information

## References

[1] Thomson JA (1998). Embryonic stem cell lines derived from human blastocysts. Science.

[2] Tanabe K, Takahashi K, Yamanaka S (2014). Induction of pluripotency by defined factors. Proceedings of the Japan Academy, Series B.

[3] Yu J (2011). Induced Pluripotent Stem Cell Lines Derived from Human Somatic Cells. Stem Cell Reviews and Reports.

[4] Takahashi K, Yamanaka S (2006). Induction of pluripotent stem cells from mouse embryonic and adult fibroblast cultures by defined factors. Cell.

[5] Takahashi K (2007). Induction of Pluripotent Stem Cells from Adult Human Fibroblasts by Defined Factors. Cell.

[6] Raya Á (2009). Disease-corrected haematopoietic progenitors from Fanconi anaemia induced pluripotent stem cells. Nature.

[7] Csete M (2010). Translational prospects for human induced pluripotent stem cells. Regenerative Medicine.

[8] Lindvall O, Kokaia Z (2010). Stem cells in human neurodegenerative disorders–time for clinical translation?. Journal of Clinical Investigation.

[9] Devine MJ (2011). Parkinson’s disease induced pluripotent stem cells with triplication of the α-synuclein locus. Nature Communications.

[10] Hossini, A. M., Megges, M., Prigione, A. & Lichtner, B. Erratum: Induced pluripotent stem cell-derived neuronal cells from a sporadic Alzheimer’s disease donor as a model for investigating AD-associated gene regulatory networks. **16**, 84 (2015). 10.1186/s12864-015-1262-5 PMC4344782 25765079

[11] Israel MA (2012). Probing sporadic and familial Alzheimer’s disease using induced pluripotent stem cells. Nature.

[12] Mahairaki V (2014). Induced pluripotent stem cells from familial Alzheimer’s disease patients differentiate into mature neurons with amyloidogenic properties. Stem Cells & Development.

[13] Richard JP, Maragakis NJ (2015). Induced pluripotent stem cells from ALS patients for disease modeling. Brain Research.

[14] Egawa N (2012). Drug screening for ALS using patient-specific induced pluripotent stem cells. Science Translational Medicine.

[15] Inoue H, Nagata N, Kurokawa H, Yamanaka S (2014). iPS cells: a game changer for future medicine. Embo Journal.

[16] Soldner F (2011). Generation of isogenic pluripotent stem cells differing exclusively at two early onset Parkinson point mutations. Cell.

[17] Park IH (2008). Disease-Specific Induced Pluripotent Stem Cells. Cell.

[18] Brennand KJ (2011). Modelling schizophrenia using human induced pluripotent stem cells. Nature. Nature.

[19] Yacoub E, Van DMPA, Ugurbil K (2013). SHANK3 and IGF1 restore synaptic deficits in neurons from 22q13 deletion syndrome patients. Nature.

[20] Karumbayaram S (2009). Directed differentiation of human induced pluripotent stem cells generates active motor neurons. Stem Cells.

[21] Chambers SM (2009). Highly efficient neural conversion of human ES and iPS cells by dual inhibition of SMAD signaling. Nature Biotechnology.

[22] Nizzardo M (2010). *Human motor neuron generation from embryon*ic stem cells and induced pluripotent stem cells. Cellular & Molecular Life Sciences Cmls.

[23] Kirwan P (2015). Development and function of human cerebral cortex neural networks from pluripotent stem cells in vitro. Development.

[24] Zhang Y (2013). Rapid Single-Step Induction of Functional Neurons from Human Pluripotent Stem Cells. Neuron.

[25] Bardy C (2015). Neuronal medium that supports basic synaptic functions and activity of human neurons in vitro. Proceedings of the National Academy of Sciences of the United States of America.

[26] Chanda (2014). Generation of Induced Neuronal Cells by the Single Reprogramming Factor ASCL1. Stem Cell Reports.

[27] Bardy, C. et al. Predicting the functional states of human iPSC-derived neurons with single-cell RNA-seq and electrophysiology. *Molecular Psychiatry* (2016). 10.1038/mp.2016.158 PMC5071135 27698428

[28] Keshishian HRH’s (2004). “The outgrowth of the nerve fiber as a mode of protoplasmic movement”. Journal of Experimental Zoology Part A Comparative Experimental Biology.

[29] Leonardo DA (2014). Large-scale generation of human iPSC-derived neural stem cells/early neural progenitor cells and their neuronal differentiation. Organogenesis.

[30] Prè D (2014). A time course analysis of the electrophysiological properties of neurons differentiated from human induced pluripotent stem cells (iPSCs). Plos One.

[31] Korkut C, Budnik V (2009). WNTs tune up the neuromuscular junction. Nature Reviews Neuroscience.

[32] Li HL (2015). Precise correction of the dystrophin gene in duchenne muscular dystrophy patient induced pluripotent stem cells by TALEN and CRISPR-Cas9. Stem Cell Reports.

[33] Stelzl U (2005). A human protein-protein interaction network: a resource for annotating the proteome. Cell.

[34] Thomas R (2014). A proteome-scale map of the human interactome network. Cell.

[35] Shannon P (2003). Cytoscape: A Software Environment for Integrated Models of Biomolecular Interaction Networks. Genome research.

