# Supplementary material for: Characteristic analyses of a neural differentiation model from iPSC-derived neuron according to morphology, physiology, and global gene expression pattern
Source: Sci Rep. 2017 Sep 25;7:12233. doi: 10.1038/s41598-017-12452-x (PMC5612987; doi:10.1038/s41598-017-12452-x)
Supplement: Supplementary file 1 — Supplementary Information [file 41598_2017_12452_MOESM1_ESM.doc]

**Characteristic analyses of a neural differentiation model from iPSC-derived neuron according to morphology, physiology, and global gene expression pattern**

**Kang Sai^#^1, Chen Xiaoxia^#^1, Gong Siyi^#^1, Yu Panpan1, YAU Suk Yu2, Su Zhenghui3, Zhou Libing1, Yu Jiandong1*, Pan Guangjing3*, Shi Lingling1,4,5***

**1 Guangdong-Hong Kong-Macau Institute of CNS Regeneration, Jinan University, Guangzhou, China.**

**2 Department of Rehabilitation Sciences, Hong Kong Polytechnic University, Hung Hom, Hong Kong SAR, China**

**3 Key Laboratory of Regenerative Biology, South China Institute for Stem Cell Biology and Regenerative Medicine, Guangzhou Institutes of Biomedicine and Health, Chinese Academy of Sciences, Guangzhou, China.**

**4 Joint International Research　Laboratory of CNS Regeneration( Jinan University), Ministry of Education, Guangzhou 510632, China.**

**5 Co-innovation Center of Neuroregeneration, Nantong University, Nantong, China.**

**^#^ These authors contributed equally to this work.**

**Correspondence and requests for materials should be addressed to**

**Shi Lingling (email: tlingshi@jnu.edu.cn) or to**

**Pan Guangjin (email: pan_guangjin@gibh.ac.cn) or to**

**Yu Jiandong (email: jiandongyu@qq.com).**

**Supplementary Information**

**Fig-S1（Shi）**

**
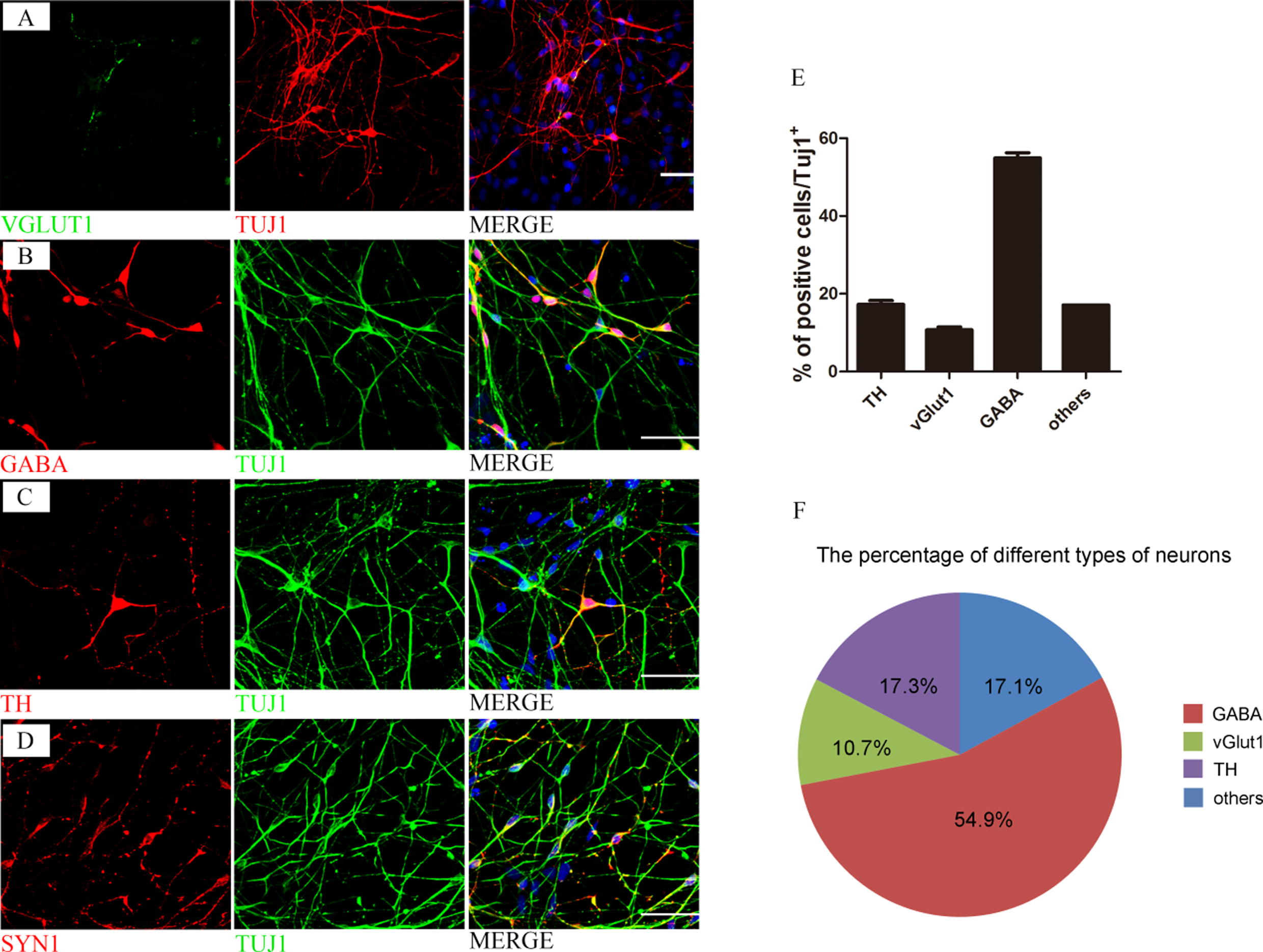
**

**Fig.S1 IPSC-derived neurons can differentiate into excitatory and inhibitory neurons.** Differentiated cell DIV10 can be stained with excitatory neuron and inhibitory neuron marker. (A) Some differentiated neurons were stained positive for vGlut1, a marker of the excitatory neuron. (B, C) Some of the differentiated neurons stained positive for GABA or TH, both of which are markers of inhibitory neurons. (D) The neurons stained positive for Synapsin1. In this differentiation protocol, 54.9% were gabaergic neurons, 10.7% were glutamatergic neurons, 17.3% were TH neurons, and 17.1% were others.

Scale bar: 200 μm.

**Fig-S2（Shi）**


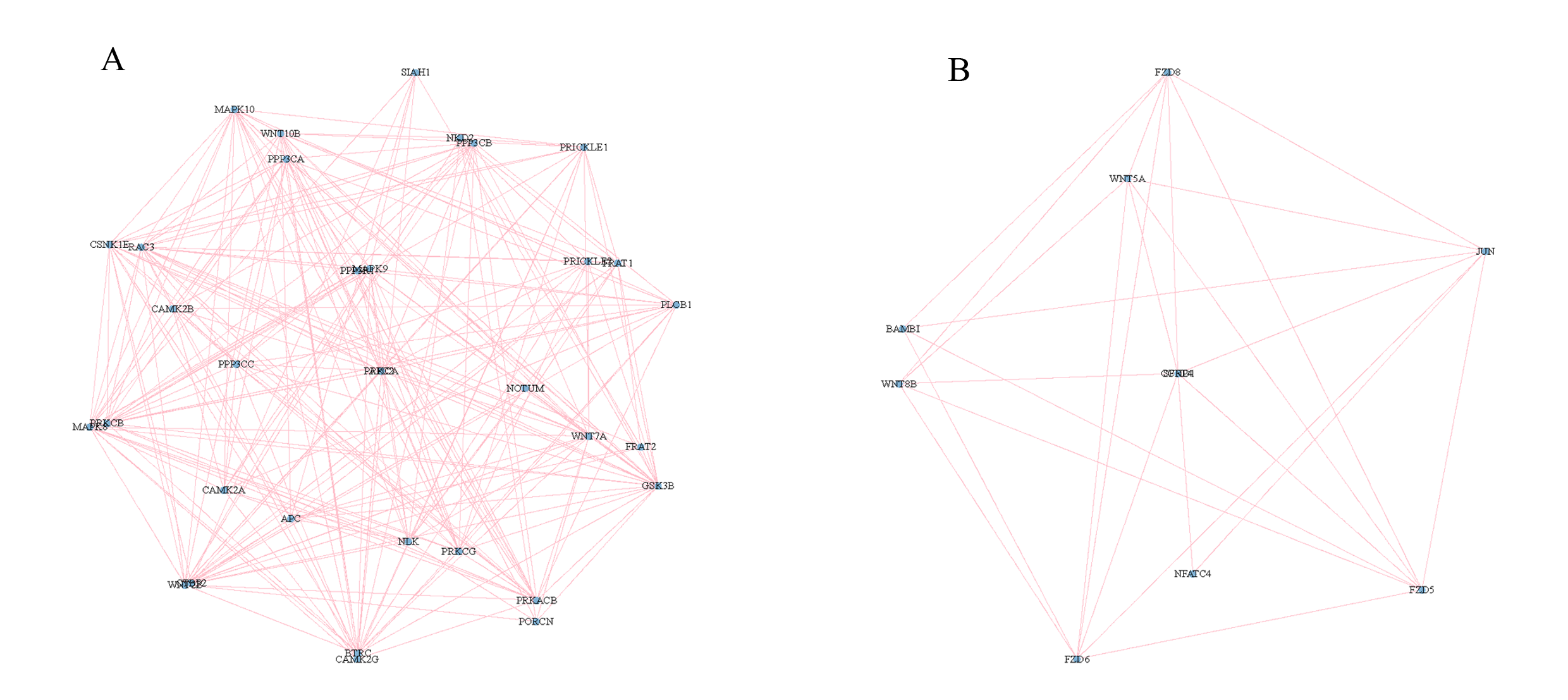


**Fig.S2 Gene-gene network of the WNT pathway-associated gene cluster. (**A) network of up regulated genes from D0 to D7 constructed by PPI framework. (B) network of up regulated genes from D7 to D28 constructed by PPI framework.

**Table S1: Top down and up pathway enrichment** from modules by GO

| GO enrichment of module 11: up regulated | | | | | | | | | | | | | | | | | | |  |
| --- | --- | --- | --- | --- | --- | --- | --- | --- | --- | --- | --- | --- | --- | --- | --- | --- | --- | --- | --- |
| Rank | | | | GO_ID | GO_Term | | | | Pvalue | | | | | | AdjustedPv | | | |  |
| 1 | | | | GO:0043005 | neuron projection | | | | 3.24E-16 | | | | | | 3.26E-12 | | | |  |
| 2 | | | | GO:0097458 | neuron part | | | | 5.07E-15 | | | | | | 2.56E-11 | | | |  |
| 3 | | | | GO:0036477 | Soma to dendritic compartment | | | | 1.54E-14 | | | | | | 5.18E-11 | | | |  |
| 4 | | | | GO:0030425 | dendrite | | | | 5.01E-12 | | | | | | 8.41E-09 | | | |  |
| 5 | | | | GO:0031175 | neuron projection development | | | | 4.65E-10 | | | | | | 5.58E-07 | | | |  |
| 6 | | | | GO:0007268 | synaptic transmission | | | | 5.68E-10 | | | | | | 5.58E-07 | | | |  |
| 7 | | | | GO:0045202 | synapse | | | | 6.09E-10 | | | | | | 5.58E-07 | | | |  |
| 8 | | | | GO:0044456 | synapse part | | | | 7.67E-09 | | | | | | 3.86E-06 | | | |  |
| 9 | | | | GO:0042995 | cell projection | | | | 8.44E-09 | | | | | | 3.98E-06 | | | |  |
| 10 | | | | GO:0006914 | autophagy | | | | 1.17E-08 | | | | | | 5.13E-06 | | | |  |
| GO enrichment of module 12: up regulated | | | | | | | | | | | | | | | | | | | |
| Rank | | GO_ID | | | | | | GO_Term | | | | | | Pvalue | | | | AdjustedPv | |
| 1 | | GO:0031410 | | | | | | cytoplasmic vesicle | | | | | | 3.55E-07 | | | | 0.002189 | |
| 2 | | GO:0016023 | | | | | | cytoplasmic membrane-bounded vesicle | | | | | | 1.54E-06 | | | | 0.004749 | |
| 3 | | GO:0044433 | | | | | | cytoplasmic vesicle part | | | | | | 1.21E-05 | | | | 0.024949 | |
| 4 | | GO:0030141 | | | | | | secretory granule | | | | | | 2.02E-05 | | | | 0.031217 | |
| 5 | | GO:0006690 | | | | | | icosanoid metabolic process | | | | | | 0.000128 | | | | 0.073216 | |
| 6 | | GO:1901568 | | | | | | fatty acid derivative metabolic process | | | | | | 0.000128 | | | | 0.073216 | |
| 7 | | GO:0010936 | | | | | | negative regulation of macrophage cytokine production | | | | | | 0.000131 | | | | 0.073216 | |
| 8 | | GO:0015672 | | | | | | monovalent inorganic cation transport | | | | | | 0.000176 | | | | 0.083415 | |
| 9 | | GO:0090066 | | | | | | regulation of anatomical structure size | | | | | | 0.000191 | | | | 0.083668 | |
| 10 | | GO:0012506 | | | | | | vesicle membrane | | | | | | 0.000206 | | | | 0.083668 | |
| GO enrichment of module 13: up regulated | | | | | | | | | | | | | | | | | | | |
| Rank | | | | GO_ID | | | GO_Term | | | | P value | | | | | AdjustedPv | | | |
| 1 | | | | GO:0022838 | | | substrate-specific channel activity | | | | 2.16E-08 | | | | | 7.95E-05 | | | |
| 2 | | | | GO:0006820 | | | anion transport | | | | 2.63E-08 | | | | | 7.95E-05 | | | |
| 3 | | | | GO:0015698 | | | inorganic anion transport | | | | 3.43E-08 | | | | | 7.95E-05 | | | |
| 4 | | | | GO:0022803 | | | passive transmembrane transporter activity | | | | 8.38E-08 | | | | | 0.000145 | | | |
| 5 | | | | GO:0005216 | | | ion channel activity | | | | 1.25E-07 | | | | | 0.000145 | | | |
| 6 | | | | GO:0015075 | | | ion transmembrane transporter activity | | | | 1.53E-07 | | | | | 0.000152 | | | |
| 7 | | | | GO:0022891 | | | substrate-specific transmembrane transporter activity | | | | 2.94E-07 | | | | | 0.000214 | | | |
| 8 | | | | GO:0015267 | | | channel activity | | | | 3.08E-07 | | | | | 0.000214 | | | |
| 9 | | | | GO:0031226 | | | intrinsic component of plasma membrane | | | | 3.65E-07 | | | | | 0.000231 | | | |
| 10 | | | | GO:0022857 | | | transmembrane transporter activity | | | | 4.66E-07 | | | | | 0.00027 | | | |
| GO enrichment of module 15: up regulated | | | | | | | | | | | | | | | | | | | |
| Rank | | | | GO_ID | | | GO_Term | | | | Pvalue | | | | | AdjustedPv | | | |
| 1 | | | | GO:0044459 | | | plasma membrane part | | | | 3.74E-05 | | | | | 0.015278 | | | |
| 2 | | | | GO:0044456 | | | synapse part | | | | 7.45E-05 | | | | | 0.028736 | | | |
| 3 | | | | GO:0019200 | | | carbohydrate kinase activity | | | | 9.01E-05 | | | | | 0.032924 | | | |
| 4 | | | | GO:0071805 | | | potassium ion transmembrane transport | | | | 0.000124 | | | | | 0.041148 | | | |
| 5 | | | | GO:0071804 | | | cellular potassium ion transport | | | | 0.000124 | | | | | 0.041148 | | | |
| 6 | | | | GO:0046835 | | | carbohydrate phosphorylation | | | | 0.000162 | | | | | 0.048929 | | | |
| 7 | | | | GO:0007193 | | | adenylate cyclase-inhibiting G-protein coupled receptor signaling pathway | | | | 0.00032 | | | | | 0.085542 | | | |
| 8 | | | | GO:0032225 | | | regulation of synaptic transmission, dopaminergic | | | | 0.000397 | | | | | 0.100649 | | | |
| 9 | | | | GO:0019203 | | | carbohydrate phosphatase activity | | | | 0.00042 | | | | | 0.100649 | | | |
| 10 | | | | GO:0050308 | | | sugar-phosphatase activity | | | | 0.00042 | | | | | 0.100649 | | | |
| GO enrichment of module 2: down regulated | | | | | | | | | | | | | | | | | | | |
| Rank | | | GO_ID | | | GO_Term | | | | | | | Pvalue | | | | AdjustedPv | | |
| 1 | | | GO:0006139 | | | nucleobase-containing compound metabolic process | | | | | | | 1.98E-07 | | | | 0.000349 | | |
| 2 | | | GO:0046483 | | | heterocycle metabolic process | | | | | | | 2.04E-07 | | | | 0.000349 | | |
| 3 | | | GO:0006725 | | | cellular aromatic compound metabolic process | | | | | | | 1.27E-06 | | | | 0.00107 | | |
| 4 | | | GO:1901360 | | | organic cyclic compound metabolic process | | | | | | | 1.72E-06 | | | | 0.00107 | | |
| 5 | | | GO:0034641 | | | cellular nitrogen compound metabolic process | | | | | | | 1.83E-06 | | | | 0.00107 | | |
| 6 | | | GO:0018130 | | | heterocycle biosynthetic process | | | | | | | 2.11E-06 | | | | 0.00107 | | |
| 7 | | | GO:0019438 | | | aromatic compound biosynthetic process | | | | | | | 2.20E-06 | | | | 0.00107 | | |
| 8 | | | GO:0034654 | | | nucleobase-containing compound biosynthetic process | | | | | | | 2.58E-06 | | | | 0.00107 | | |
| 9 | | | GO:0044271 | | | cellular nitrogen compound biosynthetic process | | | | | | | 2.81E-06 | | | | 0.00107 | | |
| 10 | | | GO:1901362 | | | organic cyclic compound biosynthetic process | | | | | | | 3.27E-06 | | | | 0.001121 | | |
| GO enrichment of module 3: down regulated | | | | | | | | | | | | | | | | | | | |
| Rank | | | | GO_ID | | | GO_Term | | | | Pvalue | | | | | AdjustedPv | | | |
| 1 | | | | GO:0006397 | | | mRNA processing | | | | 2.18E-06 | | | | | 0.004337 | | | |
| 2 | | | | GO:0003723 | | | RNA binding | | | | 2.73E-06 | | | | | 0.004337 | | | |
| 3 | | | | GO:0006396 | | | RNA processing | | | | 3.42E-05 | | | | | 0.019899 | | | |
| 4 | | | | GO:0016866 | | | intramolecular transferase activity | | | | 0.000167 | | | | | 0.059029 | | | |
| 5 | | | | GO:0016071 | | | mRNA metabolic process | | | | 0.000455 | | | | | 0.131592 | | | |
| 6 | | | | GO:0044822 | | | poly(A) RNA binding | | | | 0.0006 | | | | | 0.149213 | | | |
| 7 | | | | GO:0006457 | | | protein folding | | | | 0.00061 | | | | | 0.149213 | | | |
| 8 | | | | GO:0001522 | | | pseudouridine synthesis | | | | 0.000683 | | | | | 0.155048 | | | |
| 9 | | | | GO:0008380 | | | RNA splicing | | | | 0.000768 | | | | | 0.162627 | | | |
| 10 | | | | GO:0003676 | | | nucleic acid binding | | | | 0.000846 | | | | | 0.167962 | | | |
| GO enrichment of module 1: disorder | | | | | | | | | | | | | | | | | | | |
| Rank | | | | GO_ID | | | GO_Term | | | Pvalue | | | | | | AdjustedPv | | | |
| 1 | | | | GO:0044422 | | | organelle part | | | 2.00E-26 | | | | | | 2.36E-22 | | | |
| 2 | | | | GO:0044446 | | | intracellular organelle part | | | 5.66E-26 | | | | | | 3.33E-22 | | | |
| 3 | | | | GO:0043226 | | | organelle | | | 5.67E-25 | | | | | | 2.22E-21 | | | |
| 4 | | | | GO:0044444 | | | cytoplasmic part | | | 1.64E-23 | | | | | | 4.81E-20 | | | |
| 5 | | | | GO:0044424 | | | intracellular part | | | 5.40E-23 | | | | | | 1.27E-19 | | | |
| 6 | | | | GO:0043227 | | | membrane-bounded organelle | | | 1.65E-22 | | | | | | 3.23E-19 | | | |
| 7 | | | | GO:0005622 | | | intracellular | | | 2.35E-22 | | | | | | 3.95E-19 | | | |
| 8 | | | | GO:0005737 | | | cytoplasm | | | 3.22E-22 | | | | | | 4.74E-19 | | | |
| 9 | | | | GO:0043229 | | | intracellular organelle | | | 6.25E-22 | | | | | | 8.17E-19 | | | |
| 10 | | | | GO:0043231 | | | intracellular membrane-bounded organelle | | | 8.09E-21 | | | | | | 9.52E-18 | | | |
| GO enrichment of module 14: disorder | | | | | | | | | | | | | | | | | | | |
| Rank | GO_ID | | | | | GO_Term | | | | | | Pvalue | | | | | AdjustedPv | | |
| 1 | GO:0044456 | | | | | synapse part | | | | | | 2.46E-24 | | | | | 1.40E-20 | | |
| 2 | GO:0097458 | | | | | neuron part | | | | | | 3.08E-24 | | | | | 1.40E-20 | | |
| 3 | GO:0045202 | | | | | synapse | | | | | | 3.82E-24 | | | | | 1.40E-20 | | |
| 4 | GO:0007268 | | | | | synaptic transmission | | | | | | 8.93E-23 | | | | | 2.46E-19 | | |
| 5 | GO:0043005 | | | | | neuron projection | | | | | | 1.07E-20 | | | | | 2.35E-17 | | |
| 6 | GO:0048666 | | | | | neuron development | | | | | | 2.65E-17 | | | | | 4.86E-14 | | |
| 7 | GO:0007399 | | | | | nervous system development | | | | | | 3.61E-17 | | | | | 5.68E-14 | | |
| 8 | GO:0097060 | | | | | synaptic membrane | | | | | | 1.11E-16 | | | | | 1.53E-13 | | |
| 9 | GO:0030182 | | | | | neuron differentiation | | | | | | 5.63E-15 | | | | | 6.89E-12 | | |
| 10 | GO:0031175 | | | | | neuron projection development | | | | | | 6.58E-15 | | | | | 7.25E-12 | | |

**Table S2:** Combined WNTs gene expression with WNT signal pathway

| Stage | canonical WNT pathway | | | noncanonical WNT pathway | |
| --- | --- | --- | --- | --- | --- |
|  | WNT5A | FZD | β-Catenin | WNT7A | NLK |
| D0-D7 | down | down | down | up | up |
| D7-D28 | up | up | up | down | down |
